# Neurons Export Extracellular Vesicles Enriched in Cysteine String Protein and Misfolded Protein Cargo

**DOI:** 10.1038/s41598-017-01115-6

**Published:** 2017-04-19

**Authors:** Jingti Deng, Carolina Koutras, Julien Donnelier, Mana Alshehri, Maryam Fotouhi, Martine Girard, Steve Casha, Peter S. McPherson, Stephen M. Robbins, Janice E. A. Braun

**Affiliations:** 1 Hotchkiss Brain Institute, Department of Biochemistry and Molecular Biology, Cumming School of Medicine, University of Calgary, Calgary, Alberta Canada; 2 Southern Alberta Cancer Research Institute, Department of Biochemistry and Molecular Biology, Cumming School of Medicine, University of Calgary, Calgary, Alberta Canada; 3 Montreal Neurological Institute, Department of Neurology and Neurosurgery, McGill University, Montreal, Quebec Canada; 4 Hotchkiss Brain Institute, Department of Clinical Neurosciences, Cumming School of Medicine, University of Calgary, Calgary, Alberta Canada

## Abstract

The fidelity of synaptic transmission depends on the integrity of the protein machinery at the synapse. Unfolded synaptic proteins undergo refolding or degradation in order to maintain synaptic proteostasis and preserve synaptic function, and buildup of unfolded/toxic proteins leads to neuronal dysfunction. Many molecular chaperones contribute to proteostasis, but one in particular, cysteine string protein (CSPα), is critical for proteostasis at the synapse. In this study we report that exported vesicles from neurons contain CSPα. Extracellular vesicles (EV’s) have been implicated in a wide range of functions. However, the functional significance of neural EV’s remains to be established. Here we demonstrate that co-expression of CSPα with the disease-associated proteins, polyglutamine expanded protein 72Q huntingtin^ex^°^n1^ or superoxide dismutase-1 (SOD-1^G93A)^ leads to the cellular export of both 72Q huntingtin^ex^°^n1^ and SOD-1^G93A^ via EV’s. In contrast, the inactive CSPα_HPD-AAA_ mutant does not facilitate elimination of misfolded proteins. Furthermore, CSPα-mediated export of 72Q huntingtin^ex^°^n1^ is reduced by the polyphenol, resveratrol. Our results indicate that by assisting local lysosome/proteasome processes, CSPα-mediated removal of toxic proteins via EVs plays a central role in synaptic proteostasis and CSPα thus represents a potential therapeutic target for neurodegenerative diseases.

## Introduction

Synaptic proteostasis is the foundation of functional synaptic networks. Neurons have numerous presynaptic terminals that operate with high frequency at a great distance from the cell body and there are significant demands on the protein folding capacity at the synapse. Imbalances between the protein folding load and folding capacity result in accumulation of misfolded, nonfunctional proteins and protein aggregates that ultimately trigger synaptic loss. Cysteine string protein (CSPα, DnaJC5) is a synaptic vesicle associated co-chaperone, enriched in synapses, that forms a complex with Hsp70 family members on synaptic vesicles to facilitate presynaptic protein folding^1, 2^. Many Hsp70-interacting proteins, such as CSPα, contain a ~70 amino acid -J domain. Within the J domain of CSPα, the HPD motif is required for the interaction and activation of the Hsp70 family member^3^. Other mutations in CSPα, L115R and L116∆, cause the neurodegenerative disorder, adult neuronal ceroid lipofuscinosis (ANCL) emphasizing the critical role for CSPα in maintaining synapse function^4–6^. Moreover, deletion of CSPα leads to neurodegeneration in multiple experimental models. CSPα knock out mice exhibit fulminant neurodegeneration and have a reduced lifespan with no mice surviving beyond 3 months. In contrast, CSPα^+/−^ mice are for the most part phenotypically normal, suggesting that there is a surplus of the co-chaperone under normal physiological conditions^7–9^. Loss-of-function CSPα *Drosophila* mutants demonstrate uncoordinated movements, temperature-sensitive paralysis and early lethality^10^. Furthermore, in *C elegans*, CSPα null mutants show neurodegeneration and reduced lifespan^11^. Clearly, dysruption or absence of CSPα has undesired consequences on the stability of synapses; nevertheless, questions remain regarding the mechanisms underlying CSPα-neuroprotection.

Some J domain-containing co-chaperones undergo secretion; DnaJB11 (Erdj3) is reported to be released from HEK-293 cells through the canonical ER/Golgi secretory pathway and DnaJB1 (Hsp40) is released from Neuro2A cells via extracellular vesicles (EV’s)^12, 13^. DnaJB1 is a cytosolic co-chaperone that is upregulated during the heat shock response^14, 15^ and is implicated in protection of the nervous system from damage^14^. Microvesicles and exosomes are EV’s that shuttle specific cargo such as proteins, lipid and RNA between cells^16^. They are released by all cell types and mediate the transfer of hydrophobic and cytosolic proteins, exerting profound effects on recipient cells and influencing proteostasis. Microvesicles are large EV’s, ~100 nm-1 μm in diameter, formed by the outward budding of the plasma membrane. Exosomes are smaller vesicles, ~40–130 nm in diameter, formed by the inward budding of the endosomal membrane to generate intraluminal vesicles that accumulate in multivesicular bodies (MVB’s). MVB’s fuse with either the plasma membrane to release exosomes or with lysosomes for degradation of the vesicles and protein contents^17^. Exosome-exported DnaJB1 contributes to the protein folding capacity of recipient cells (eg. reducing polyglutamine-mediated aggregation in neuroblastoma cells)^13^. In contrast, DnaJB11 is an ER protein that is upregulated during the unfolded protein response^18^ and is responsible for folding and degradation of a number of ER client proteins^18–22^. DnaJB11 is implicated in neural development^23^ as well as Gaucher’s disease^22^. Several chaperones, like DnaJB11, are secreted into the extracellular space where they bind misfolded proteins^24^ and contribute to extracellular folding capacity (eg. reducing the effect of toxic prion protein on neuroblastoma cells)^12^. For example, clustrin is implicated in Alzheimer’s disease and prevents aggregation of extracellular misfolded proteins in an Hsc70 and ATP-independent manner^24^. However, a comprehensive list of extracellular chaperones surrounding synapses has not been established. In addition to enhancing recipient cell or extracellular folding capacity, it is likely that Hsp70-interacting proteins stabilize client protein delivery through the secretory pathway or during EV-transit to recipient cells.

Distinct export routes for misfolded proteins also exist. Accumulation of misfolded-toxic proteins between cells is a common pathogenic pathway^24^. For example, a major hallmark of Alzheimer’s disease is the formation of extracellular plaques that contain aggregated, amyloid β peptide^25^. In contrast, cell-to-cell transmission of misfolded-toxic proteins such as prions^26, 27^, beta-amyloid peptides^28^, amyloid precursor protein fragments^29^, tau^30, 31^ superoxide dismutase 1^32–34^ TDP-43^35^ and α-synuclein^36–38^ is EV-mediated. Many questions remain regarding how chaperones target and process exported misfolded proteins.

The full nature of the events governing presynaptic protein homeostasis is not understood. One possible scenario for CSPα’s neuroprotective activity is targeting of misfolded client proteins by CSPα for export, thereby reducing the protein-folding load in neurons. To begin to investigate this possibility we first sought to determine if CSPα is secreted from neurons. We report that CSPα is indeed exported from catecholaminergic-derived mouse (CAD) cells within extracellular vesicles (EV’s). We show that CSPα is released from mouse brain tissue as well as human glioblastoma cells and is found in human CSF. The human mutations CSPα_L115R_ and CSPα_∆116_, and the CSPα_HPD-AAA_ mutant that does not activate Hsp70, also undergo EV-mediated export. We also find that CSPα but not CSPα_HPD-AAA_, promotes cellular export of two distinct misfolded disease-causing proteins, the polyglutamine expanded protein 72Q huntingtin^exon1^ and superoxide dismutase-1 (SOD-1^G93A^). In contrast, the export of other EV components, eg. G_αs_, clathrin heavy chain, flotillin-1, Hsp90 and actin, do not show CSPα-mediated secretion. This work showing CSPα-mediated export of 72Q huntingtin^exon1^ and SOD-1^G93A^ is consistent with Fontaine and colleagues who, during the course of this study, reported that CSPα facilitated extracellular release of TDP-43, α-synuclein and microtubule-associated protein tau from HEK-293 cells^39^. Surprisingly, we find that the endogenous chaperones, DnaJB1, DnaJB11 and DnaJA1, are co-secreted with CSPα, giving rise to the possibility that several J domain-containing proteins are involved in export. Our study directly demonstrates that CSPα disposes of the misfolded-disease-causing proteins SOD-1^G93A^ and 72Q huntingtin^exon1^ by promoting their export. Given that CSPα expression is required for the survival of synapses *in vivo*, we suggest that this CSPα-mediated export is critical for the maintenance of synapses.

## Results

### CSPα is secreted from neurons

**Figure 1:**
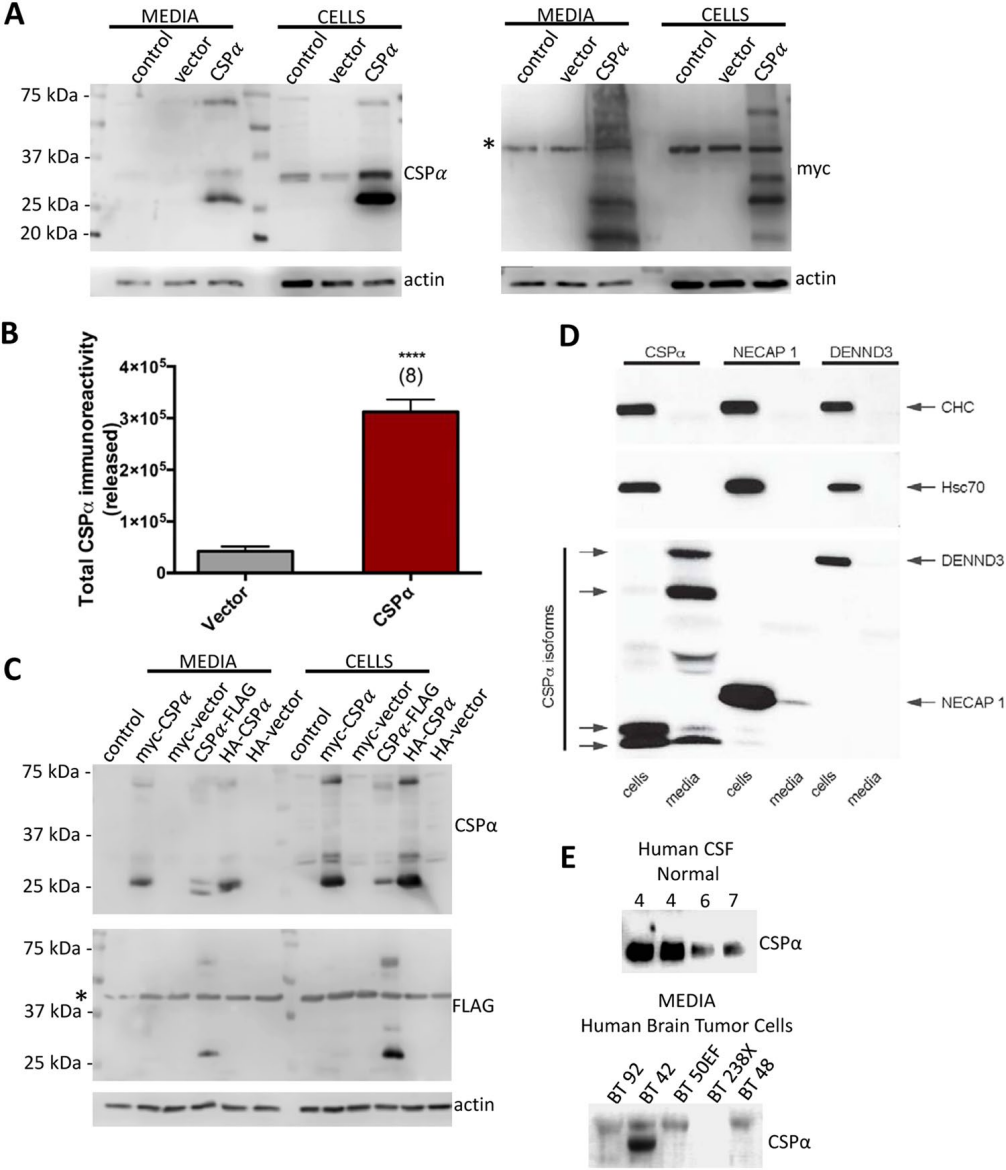
Extracellular release of CSPα. (**A**) Representative immunoblot of media and cell lysates from untransfected, vector and CSPα transfected cells. CAD cells transfected with 3.75 μg of cDNA encoding myc-tagged WT CSPα or vector control were lysed 48 hrs post-transfection and corresponding cell lysates (15 µg) and 10X concentrated media (40 µl) were evaluated by immunoblot with the indicated antibodies. *Indicates a non-specific band. (**B**) Quantification of immunoreative CSPα from vector and CSPα transfected cells (****P < 0.0001) as determined by anti-myc monoclonal. These results are representative of 8 independent experiments. (**C**) Immunoblot of lysates and media from CAD cells transfected with myc-, HA-, or FLAG-tagged CSPα and lysed 48 hrs post-transfection (**D**) Immunoblot analysis of cell lysates and culture media from HEK-293 cells transfected with Flag-CSPα, Flag-NECAP 1 or Flag-DENND3, as indicated. The bottom transfer is blotted with antibody against Flag. The top two transfers are blotted with antibodies recognizing endogenous clathrin-heavy chain (CHC) or Hsc70, as indicated. (**E**) Immunoblot of human cerebrospinal fluid (50 µl) and media from human brain tumor cell lines (75 μl).

CSPα is a well-established component of the presynaptic proteostasis network^7, 10, 40^. To begin to address whether CSPα’s neuroprotective activity involves its export, we sought to establish if CSPα is released from neurons like DnaJB1 ^13^ and DnaJB11 ^12^. To this end, myc-tagged CSPα was expressed in CAD cells, the culture media was replaced with fresh media 6 hrs after transfection and at 48 hrs, the medium was collected, centrifuged (1,500Xg) to remove dead cells and the presence of exported CSPα determined. Figure 1 shows three exported species of CSPα, consistent with the unpalmitoylated (26 kDa), palmitoylated (35 kDa) and dimeric (70 kDa) cellular forms^41, 42^ and an additional 18 kDa N terminal CSPα fragment. Actin, is a known exosome protein and is present in the conditioned media^43^. Exported CSPα is approximately 11 times higher in media from CSPα transfected CAD cells compared with vector transfected control cells, demonstrating that increasing cellular CSPα levels increases its secretion (Fig. 1B). N terminal HA- or myc- and C terminal FLAG- tagged CSPα are all released from cells (Fig. 1C), indicating that epitope tags do not influence CSPα’s export, although the C terminal FLAG tag reduced immunodetection by the CSPα polyclonal antibody. In addition to CAD cells, transiently expressed FLAG-tagged-CSPα but not Flag-NECAP or Flag-DENND3 was released from HEK-293 cells (Fig. 1D). Next we wanted to know if extracellular CSPα is a feature of transient expression, or if CSPα secretion is a more widely occurring event. To this end, we evaluated seven human CSF subjects who had no clinical evidence of CNS pathology for CSPα. All seven CSF samples evaluated were positive for CSPα, although the level of CSPα (in 50 μl) varied among subject samples (Fig. 1E and data not shown). We also evaluated CSPα levels in conditioned media from human brain tumors of 11 distinct origins^44, 45^. Out of 11 brain tumor initiating cells examined, 9 were found to secrete CSPα. CSPα of a distinct molecular weight was identified in conditioned media from two separate human brain tumors (Fig. 1E, lower panel, lane 2 and data not shown). Together, these results confirm that export of CSPα occurs physiologically, pathologically as well as in cell culture.

### CSPα is released in EV’s

**Figure 2:**
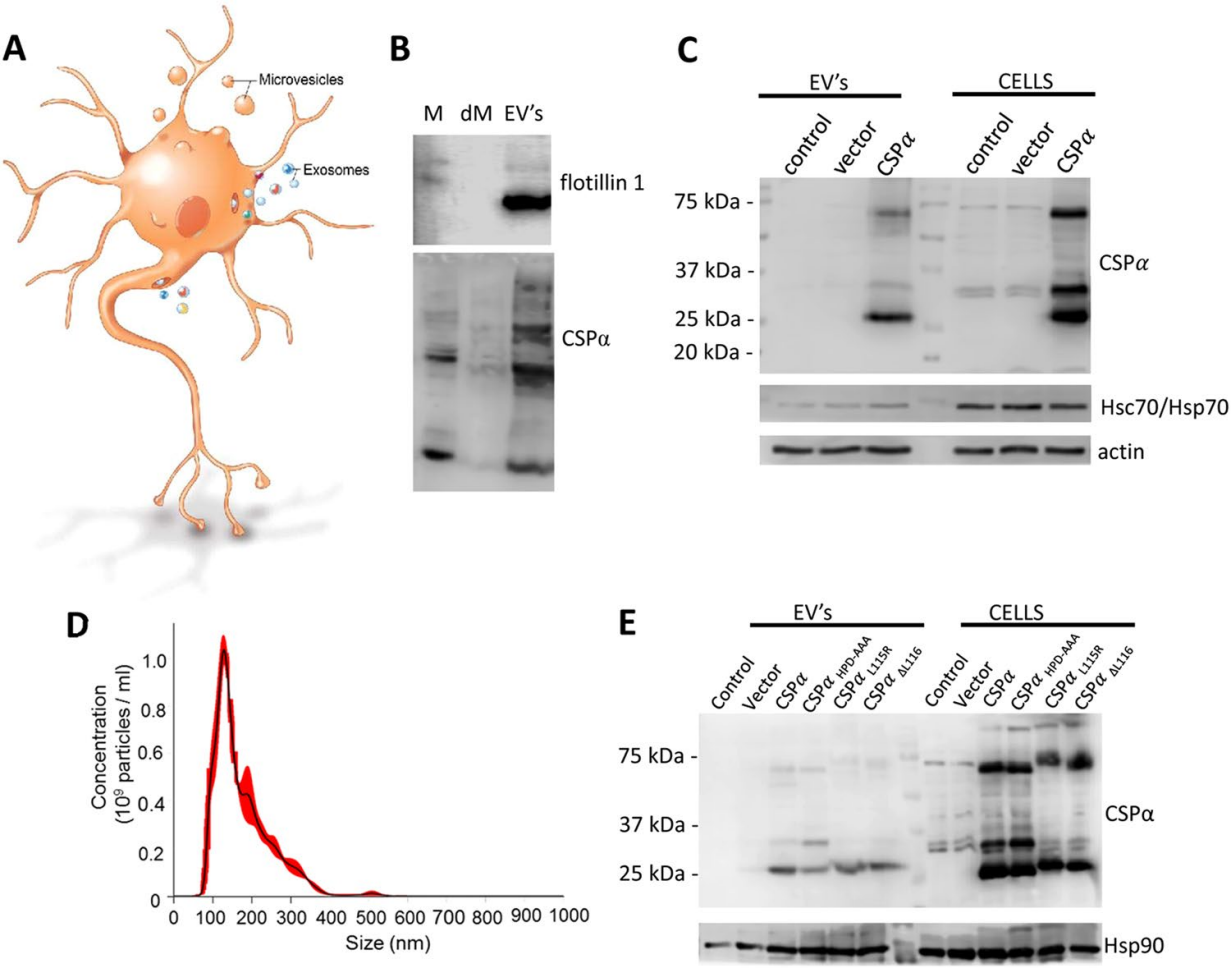
CSPα is released via EV’s. (**A**) Working model of extracellular vesicle release from neurons; illustration by Roula Drossis. Neural export of larger microvesicles and smaller exosomes are shown. (**B**) Immunoblot of media (M), media depleted of EV’s (dM) and the EV’s enriched for exosomes from CSPα transfected CAD cells. (**C**) Immunoblot of EV’s and corresponding cell lysates. (**D**) Size distribution of EV’s isolated from CSPα-transfected CAD cells. Red error bars indicate +/−1 standard error of the mean. (**E**) Immunoblot of EV’s and CAD cell lysates (15 µg) collected 48 hrs after transfection of 3.7 μg of vector, myc-tagged CSPα, or 5 μg of myc-tagged CSPα_L115R_, CSPα_L116∆_, CSPα_HPD-AAA._ Western blots are representative of 5 independent experiments.

Since conditioned medium contains secreted proteins and different types of extracellular vesicles; including exosomes and microvesicles (Fig. 2A), we next established the nature of exported CSPα. CSPα is one of the most highly palmitoylated neural proteins^46^ and we reasoned it could be secreted in exosomes that are derived from lipid rafts. Following the established ExoQuick precipitation technique (System Biosciences Inc), we isolated EV’s enriched in exosomes from cell culture media that had been centrifuged to remove cell debris. Western blot analysis reveals that CSPα is present in EV’s enriched for exosomes together with the exosomal marker flotillin 1 (Fig. 2B). Actin and Hsc70/Hsp70, known exosomal proteins^43^, are also present in these CSPα-containing EV’s (Fig. 2C). No large apoptotic bodies were present in the EV preparation. The observed EV size distribution in our preparations varied, with the most frequent size of EV’s being 129+/−2.2 nm, however larger EV’s were also present and the mean size of EV’s was 175.3+/−3.5 nm (Fig. 2D).

We wanted to know if mutated CSPα is also secreted. Figure 2E shows that like WT CSPα, myc-tagged CSPα_L115R_, CSPα_L116∆_ and a CSPα_HPD-AAA_ mutant that does not activate Hsp70 family members, are all released extracellularly. The pathogenesis of L115R and L116∆ CSPα mutants that cause ANCL^4–6^ is not established, however CSPα_L115R_ and CSPα_L116∆_ are predisposed to oligomerize and have reduced palmitoylation^42, 47, 48^. To examine whether these mutant CSPα’s were exported or retained by neurons, myc-tagged CSPα_L115R_ and CSPα_L116∆_ and loss-of-function CSPα_HPD-AAA_ were transfected into CAD cells and EV’s enriched for exosomes were analyzed 48 hrs later. Consistent with previous reports^42, 47, 48^, the 35 kDa palmitoylated CSPα species is significantly reduced in CSPα_L115R_, CSPα_L116∆_ expressing cells compared to cells expressing CSPα_HPD-AAA_ and WT CSPα and expression of CSPα_L115R_ and CSPα_L116∆_ results in formation of high molecular weight aggregates. These results demonstrate that like CSPα, CSPα mutants are present in EV’s enriched for exosomes. Secretion of CSPα_HPD-AAA_ indicates that export is independent of molecular interactions with Hsc70, whereas secretion of CSPα_L115R_ and CSPα_L116∆_ suggests that export is independent of palmitoylation. Hsp90, an established exosomal protein^43^, is present in all EV’s (Fig. 2E).

### Co-secretion with CSPα

**Figure 3:**
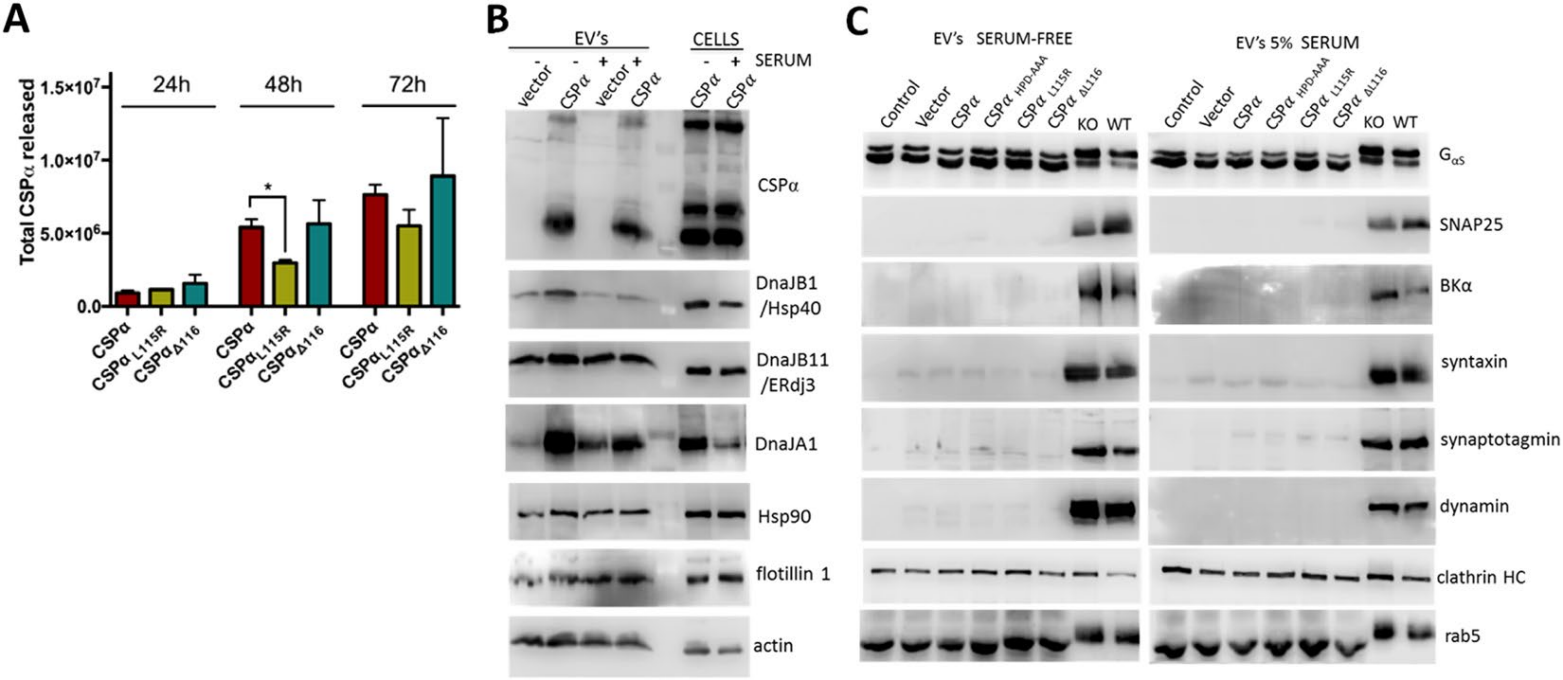
DnaJB11 and DnaJB1 are co-secreted with CSPα. (**A**) Quantification of the time course of WT and mutant CSPα release (media); (*P < 0.05). (**B**) Immunoblot of EV’s released from vector or CSPα transfected cells probed with the indicated antibodies. 6 hrs after transfection the media was replaced with either serum free media or media containing 5% serum (exosome-free) and 48 hrs after transfection media was collected and exosomes prepared. Cell lysates are shown on the left hand lanes. (**C**) Immunoblot of EV’s isolated from control (untransfected), vector (3.75 μg), myc-tagged CSPα (3.75 μg), CSPα_HPD-AAA_ (5 µg), CSPα_L115R_ (5 µg), or CSPα_L116∆_ (5 µg) transfected cells probed with the indicated antibodies. Crude synaptosomes from CSPα knock out and WT mice are shown in the right lanes. Western blots are representative of 5 independent experiments.

Since the human mutations CSPα_L115R_ and CSPα_L116∆_ lead to neurodegeneration but were secreted like WT CSPα, we wondered if WT and mutant CSPα differed in their time course of release. Figure 3 shows that CSPα_L115R_ has a significantly slower time course than CSPα at 24 hrs (P < 0.05) and that CSPα, CSPα_L115R_ and CSPα_L116∆_ release is abundant 48 hrs after transfection. The presence of FBS (5% exosome-free) did not alter CSPα secretion (Fig. 3B). Surprisingly, endogenous DnaJB1, DnaJB11 and DnaJA1 were found to be co-secreted with CSPα (Fig. 3B). The presence of DnaJB1, DnaJB11 and DnaJA1 in EV’s was confirmed by LC-MS/MS (data not shown). It is possible that these J domain containing chaperones are co-packaged as EV cargo, packaged into separate vesicles or alternatively, that they are independently secreted and subsequently associate with EV’s extracellularly (i.e. attached to the EV surface). CSPα and DnaJB11 secretion does not change in the presence of serum while DnaJB1 and DnaJA1 release is reduced (Fig. 3B), highlighting differences among the secreted J proteins. Flotillin 1, Hsp90 and actin, known exosomal components, are shown. We then examined CSPα-containing EV’s for presynaptic proteins that have been proposed to be CSPα targets^40, 42, 49–66^. As previously reported, SNAP25 expression is reduced^63, 64, 67^ while BKα potassium channel levels are elevated^56^ in synaptosomes from CSPα KO mice compared to WT mice. BKα potassium channel, SNAP25, dynamin, synaptotagmin and syntaxin are not abundant in EV’s from CAD cells (Fig. 3C). The putative CSPα client proteins G_αs_, clathrin heavy chain and Rab5 were observed to be exported in the absence or presence of CSPα or CSPα mutants.

### CSPα promotes/chaperones export of disease-causing proteins

**Figure 4:**
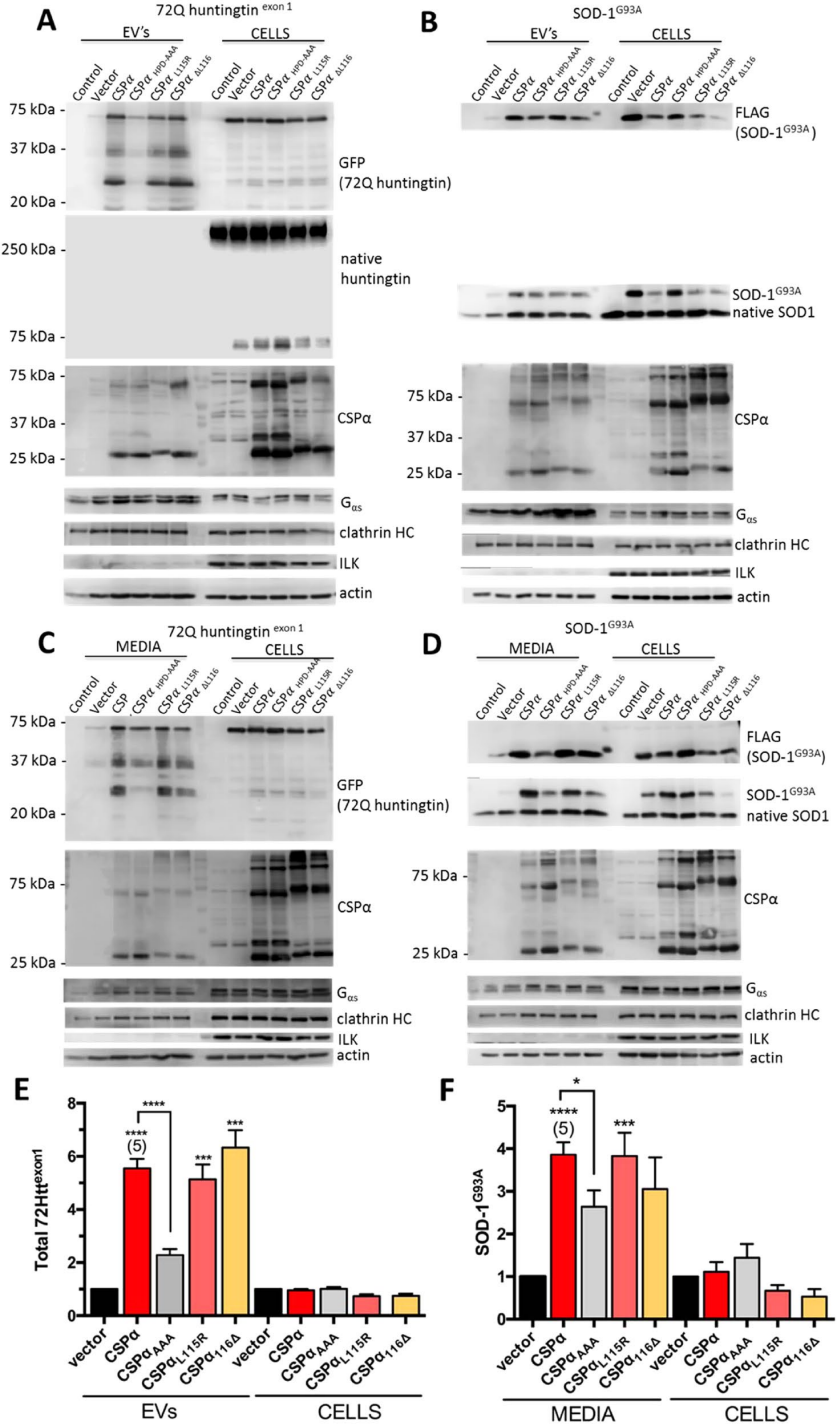
CSPα promotes 72Q huntingtin^exon1^ and superoxide dismutase-1 (SOD-1^G93A^) export. Immunoblot of EV’s and corresponding lysates (15 µg) from cells transfected with GFP-tagged 72Q huntingtin^exon1^ (**A**) or FLAG-tagged SOD-1^G93A^. For native huntingtin immunoblot 25 µg of cell lystates were evaluated. (**B**) and vector, myc-tagged CSPα, CSPα_HPD-AAA_, CSPα_L115R_, or CSPα_L116∆_ and probed with the indicated antibodies. 2.5 µg of the indicated cDNA was transfected. (**C**,**D**) Immunoblot of media and corresponding lysates (15 µg). (**E**,**F**) Quantification of GFP-tagged 72Q huntingtin^exon1^ in EV’s and FLAG-tagged SOD-1^G93A^ in media, normalized to actin (****P < 0.0001; ***P < 0.001, *P < 0.05).

We next asked if neuronal proteins associated with cellular toxicity were exported with CSPα in EV’s. To this end we introduced the polyglutamine expanded protein 72Q huntingtin^exon1^ or superoxide dismutase-1 (SOD-1^G93A^) with WT or mutant CSPα. Trinucleotide (CAG) repeat expansions of the huntingtin gene causes Huntington’s disease^68^. The length of the polyglutamine expansion is linked to the age of onset of Huntington’s disease and the aggregation propensity of the huntingtin protein^68^. Aggregates of polyglutamine expanded huntingtin have been found within genetically normal tissue grafted into patients with progressing HD, revealing cell-to-cell transit of huntingtin aggregates *in vivo* ^69^. Mutations in the gene encoding superoxide dismutase-1 (SOD-1), a copper-binding enzyme that functions as an antioxidant, lead to the motor neuron disease, amyotrophic lateral sclerosis (ALS)^70^. Misfolding, aggregation and cell-to-cell spread of mutant SOD-1 is mediated by both free floating aggregates as well as exosomes^34, 71^. Figure 4 shows that CSPα promotes cellular export of both polyglutamine expanded protein 72Q huntingtin^exon1^ and superoxide dismutase-1 (SOD-1^G93A^). CSPα_L115R_ and CSPα_L116∆_ also effectively support the export of both 72Q huntingtin^exon1^ and SOD-1^G93A.^ Integrin-linked kinase is not exported from cells while G_αs_, clathrin heavy chain, actin and native SOD-1 (lower band) are exported in EV’s in the absence and presence of CSPα and CSPα mutants. It is not known if export of these proteins is regulated by other DnaJ’s. Although export of native huntingtin from CAD cells is not observed, both WT and mutant SOD-1 are exported and export is increased by CSPα. A small increase in the cellular release of actin, clathrin heavy chain and G_αs_ is observed following transfection (vector), therefore 72Q huntingtin^exon1^ in EV’s and SOD-1^G93A^ in media was quantified relative to actin (Fig. 4E and F).

Some differences between 72Q huntingtin^exon1^ and SOD-1^G93A^ secretion were observed. EV-exported-72Q huntingtin^exon1^ underwent degradation such that 2 prominent degradation products were found in EV’s (Fig. 4A), however no SOD-1^G93A^ degradation was detected. Secreted 72Q huntingtin^exon1^ co-purified with EV’s, while secreted SOD-1^G93A^ was both free floating and EV-associated, consistent with previous reports^33^. Finally, 72Q huntingtin^exon1^ release was 60% lower (P < 0.0001) in the presence of loss-of-function CSPα_HPD-AAA_ compared to WT CSPα while SOD-1^G93A^ release, was 40% lower (P < 0.05) (Fig. 4E and F). Taken together, these results demonstrate that CSPα is exported and facilitates EV export of distinct toxic misfolded proteins. This export is dependent on the HPD motif of CSPα that is required for recruitment and activation of Hsc70 by CSPα. In contrast, the export of CSPα itself is not influenced by mutations in the HPD motif.

### CSPα is secreted from brain

**Figure 5:**
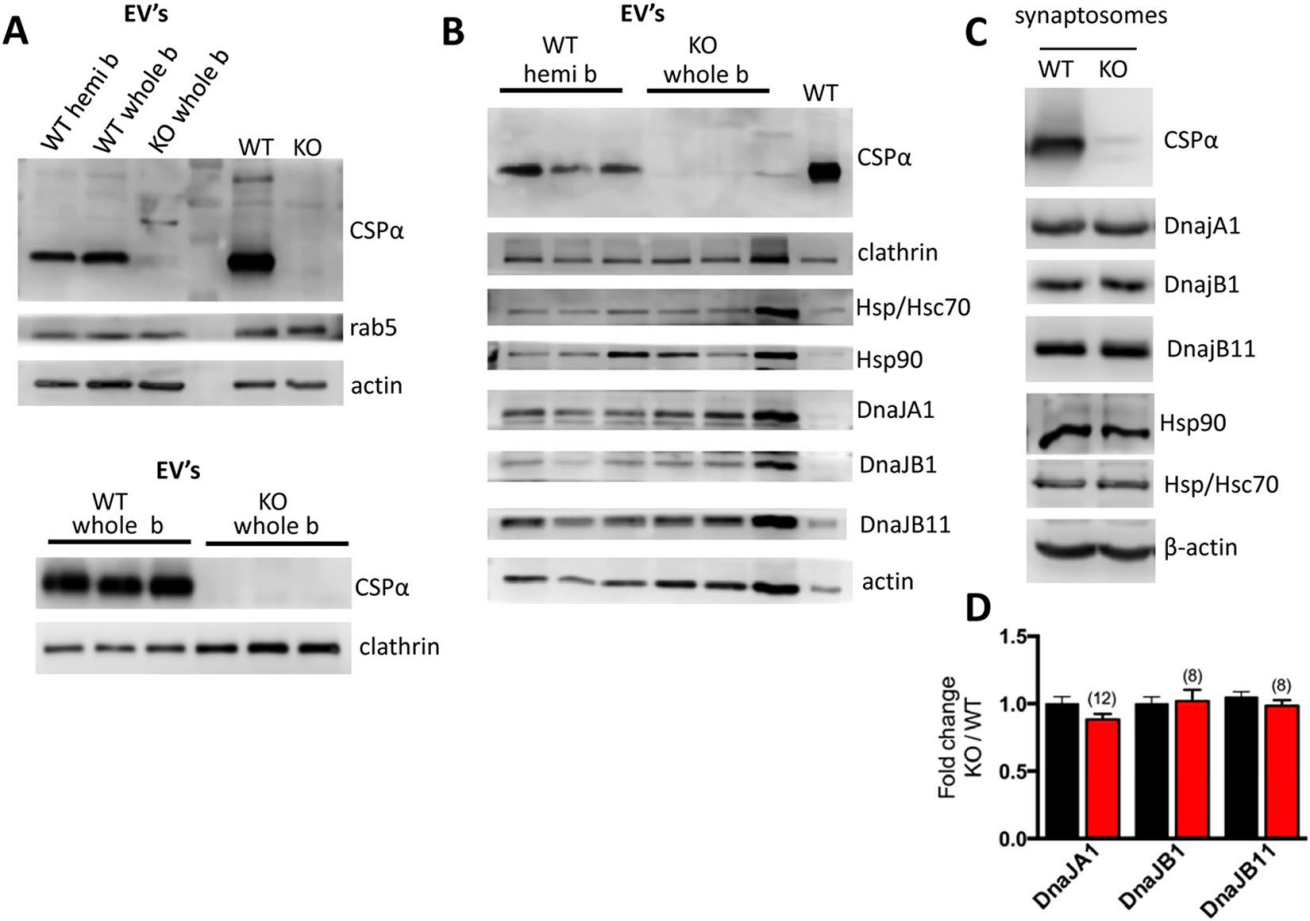
CSPα is present in EV’s isolated from brain tissues (**A**) Immunoblot of EV’s released from WT and CSPα knock out mouse brain probed with the indicated antibodies; upper panel EV’s prepared by ExoQuick and lower panel EV’s prepared by differential ultracentrifugation. Release from hemi and whole WT brains is shown for comparison. Synaptosomes (1.5 µg) from WT mice are shown in the right lane. (**B**) Immunoblot of EV’s released from WT (hemi) and CSPα knock out mouse brain (whole) probed with the indicated antibodies. (**C** and **D**) Western analysis and quantification of synaptosomes (30 µg) at 25 days probed with the indicated antibodies. β-actin is shown as loading control.

We next evaluated whether endogenous CSPα is released from mouse brain in addition to CAD cells. Brains were obtained at 23–25 days from CSPα knock out mice when they show partial paralysis and loss of neuromuscular control^7^. Mouse brains were sliced, washed in PBS, incubated in media for 2 hrs and EV’s then isolated from the supernatant that had been filtered (30µm), centrifuged (2,000Xg) to remove debris and isolated by ExoQuick precipitation. Figure 5A shows that freshly removed murine brain tissue from WT mice exports EV’s containing CSPα. As expected, no CSPα release is observed from CSPα knock out mice. CSPα is present in EV’s prepared by either ultracentrifugation or ExoQuick methods. Given the smaller size of CSPα knock out relative to WT mice^7^, we evaluated release from both whole and hemi WT brains. We asked whether or not DnaJA1, DnaJB1, and DnaJB11 were found in EV’s from mouse brain. Figure 5B shows that DnaJA1, DnaJB1, and DnaJB11 are indeed exported from both WT and CSPα knock out mouse brains. 1.5 µg of crude synaptosomes from WT mouse is shown in the right lane. No change in levels of DnaJA1, DnaJB1, DnaJB11 is found in synaptosomes from CSPα knock out mice (Fig. 5C and D). Taken together, these results indicate that CSPα and other J proteins are released from mammalian neurons and purify with EV’s.

### Resveratrol inhibits export of 72Q huntingtin^exon1^

**Figure 6:**
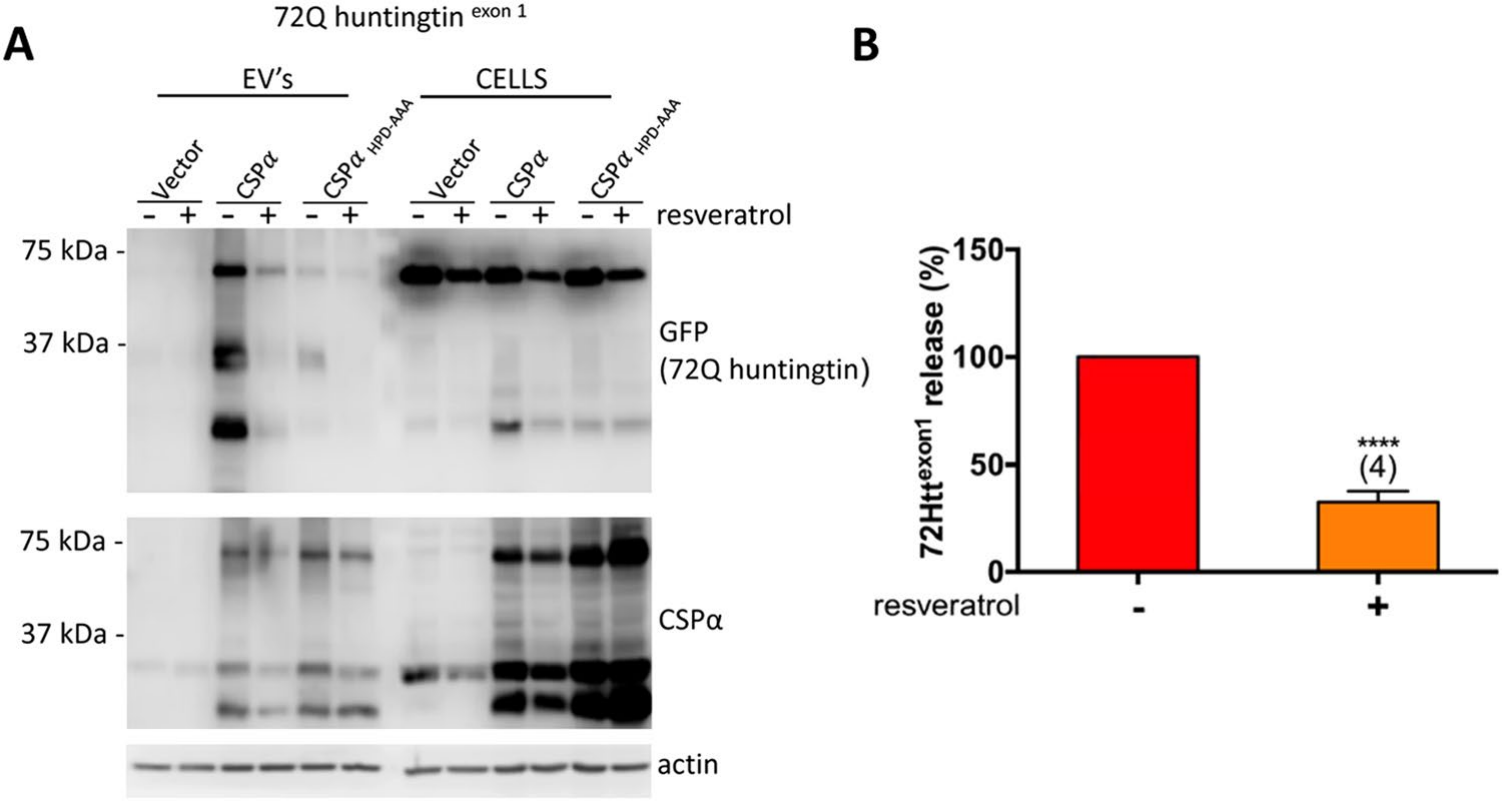
Resveratrol inhibits export of 72Q huntingtin^exon1^ (**A**) Immunoblot of EV’s and corresponding lysates (15 µg) from cells transfected with vector, CSPα or CSPα_HPD-AAA_ and GFP-tagged 72Q huntingtin. 2.5 µg of the indicated cDNA was transfected. Where indicated, cells were treated with 50 μM resveratrol 6 hrs after transfection. β-actin is shown as loading control. (**B**) Quantification of CSPα mediated export of GFP-tagged 72Q huntingtin^exon1^ in EV’s in the presence and absence of resveratrol (****p < 0.0001).

Finally we asked whether resveratrol treatment alters the CSPα promotion of disease-causing protein export. Resveratrol, a polyphenol with neurprotective, cardioprotective and anti-inflammatory properties, has been previously shown to ameliorate the reduced viability in *C. elegans* CSPα mutants^11^. Here we found that 50 µM resveratrol reduced 72Q huntingtin^exon1^ export by 70% (Fig. 6), indicating that the CSPα-mediated export of 72Q huntingtin^exon1^ is resveratrol-sensitive.

## Discussion

In this study we directly demonstrate that the neuroprotective, synaptic chaperone CSPα is secreted via EV’s and has a key role in the export of two misfolded, aggregation-prone proteins, polyglutamine expanded protein 72Q huntingtin^exon1^ and SOD-1 with a glycine to alanine amino acid substitution at position 93. Protection against the buildup of misfolded proteins that threaten synaptic function is in-line with CSPα’s well-documented neuroprotective function^7, 10^. In addition to ANCL, the rare neurodegenerative disease caused by L115R and ∆L116 mutations^4–6^, CSPα dysfunction has been implicated in a number of other neurodegenerative disorders including Alzheimer’s, Parkinson’s and Huntington’s disease however mechanistic links have not been determined^40, 42, 55, 67, 72, 73^. Our results clearly link CSPα to the motor neuron disorder ALS and polyglutamine expansion disorders like Huntington’s disease and are congruent with a recent study by Fontaine and colleagues who reported that release of TDP-43, α-synuclein and microtubule-associated protein tau from HEK-293 cells is enhanced by CSPα^39^. Together, these studies suggest the CSPα pathway is a common mechanism that promotes/chaperones export of misfolded proteins, that said, the fate of the misfolded proteins that are exported by CSPα remains to be established. The CSPα-mediated export of 72Q huntingtin^exon1^ is resveratrol-sensitive. Effective clearance of the misfolded cargo by recipient cells would represent a novel quality control pathway, on the other hand, delivery of misfolded proteins to recipient cells and subsequent propagated protein misfolding is consistent with the pathogenic prion-like spreading reported for disease-causing proteins such as, beta-amyloid, tau, TDP-43, α-synuclein, huntingtin and SOD-1. This intersection–i.e. off-site disposal vs propagated misfolding of CSPα-exported protein–is significant, and further experimentation is required to better understand these processes.

The endogenous co-secretion of DnaJA1, DnaJB1, DnaJB11 with CSPα from both CAD cells and mouse brain tissue, indicates that cellular release of “J domain”-containing proteins is more common than previously appreciated. There are many possible roles for secreted “J domain”-containing chaperones, which likely target distinct client proteins including: regulation of the assembly of the EV proteins (eg. tetraspanins, clathrin), selection of EV cargo for export, ensuring delivery of conformationally intact and functional hydrophobic and cytosolic proteins between cells, increasing the folding capacity of recipient cells via cell-to-cell transit of molecular chaperones, selection of aggregate-prone toxic proteins for elimination by export and off-site degradation, regulation of trafficking of EV’s to recipient cells and regulation of protein conformation in the extracellular space. Such processes could involve additional regulatory proteins. For example, a CSPα-interacting protein^74^, SGT (small glutamine-rich tetratricopeptide repeat containing protein) is reported to regulate whether or not misfolded proteins are targeted to late endosomes/MVBs^75^. These possible roles are based on the assumption that the secreted J proteins are indeed functional and don’t themselves represent protein trash dedicated for export. It is clear that mutated J proteins can be exported, yet it remains to be determined if exported J proteins can promote Hsp70 ATPase activity. Several J domain-containing proteins have neuroprotective activity^15^ and some, when mutated, cause neurodegenerative disease^76, 77^. For example, DnaJB6 and DnaJB2 are implicated in protection from Huntington’s disease^78^ and ALS^79^ respectively. Some J domain-containing proteins have well characterized intracellular roles; for example Sec63 (DnaJC23), is involved in protein translocation across the ER membrane^80^ and auxilin (DnaJC6), is important in uncoating clathrin coated vesicles during endocytosis^81, 82^. The fulminant neurodegeneration observed in CSPα knock-out models clearly indicates that other J proteins do not compensate in its absence.

There is currently great interest in EV’s as vehicles for intercellular communication as well as shuttles for the spread of misfolded proteins that cause neurodegenerative disorders and tumor cell metastasis. We suggest that the maintenance of optimally functional synapses involves chaperone mediated-export of dysfunctional proteins. CSPα-mediated neuroprotection is activity- dependent^8, 83^ and interestingly, release of exosomes from differentiated neurons has been reported to be regulated by synaptic activity^84^. An activity-dependent build-up of misfolded proteins may be common to the pathogenic cascade of distinct neurodegenerative diseases. Therefore, understanding export pathways is important, as it may allow for the development of therapeutics aimed at treating the progression of neurodegenerative disease.

## Materials and Methods

### Expression of chaperones, SOD-1^G93A^ and 72Q huntingtin^exon1^ in CAD cells

Maintenance of CAD (catecholaminergic derived CNS cells) was described before^56^. For expression in CAD cells, cDNAs encoding for CSPα and CSPα mutants were expressed in the plasmid myc-pCMV, HA-pCMV, pcDNA3.1-FLAG and pcDNA3.1-GFP. All mutations were confirmed by DNA sequence analysis. Constructs encoding Myc-tagged CSPα, Flag-tagged CSPα, HA-tagged Myc-tagged CSPα, Myc-tagged CSPα_L115R_, Myc-tagged CSPα_ΔL116_, Myc-tagged CSPα_HPD-AAA_, GFP-72Q huntingtin^exon1^ and SOD-1^G93A^-FLAG were transiently transfected with Lipofectamine 3000 (Invitrogen) in Opti-MEM™ medium (Invitrogen). Media was changed to serum-free media 6 hrs post-transfection, or where indicated 5% exosome free FBS (Systems Biosciences). Where indicated, 50 µM resveratrol treatment (Sigma) started 6 hrs post-transfection.

### CSF

Cerbrospinal fluid (CSF) samples were collected by lumbar puncture under general anesthesia prior to microdiskectomy surgery. The subjects were symptomatic with degenerative disc disease refractory to non-surgical management for over 12 wks but lacked evidence of CNS pathology on history and physical examination by a neurosurgeon. All the samples were collected with informed consent for participation and publication in accordance with the guidelines and regulations of the University of Calgary institutional ethics/licensing committee.

### Brain Tumor Initiating Cells

Surgical samples from patients with newly diagnosed or recurrent glioblastoma were obtained from the Tumor Tissue Bank within the Arnie Charbonneau Cancer Institute, transported to the BTIC Core Facility (Calgary, Alberta) and established as described previously^44, 45^. This study has Institutional review board approval under the “Brain Tumor and Related Tissue Bank protocol-V2” and approved by Foothills Hospital and the Conjoint Health Research Ethics Board and all methods were performed in accordance with the relevant guidelines and regulations. All established cell lines used were validated for identity by short tandem repeat analysis performed by Calgary Laboratory Services. All BTIC lines were grown in serum free-media supplemented with EGF (20 ng/ml; Peprotech, FGF2 (20 ng/ml; R&D Systems Inc, and heparin sulfate (2 mg/ml: R&D Systems). For the collection of conditioned media (CM) individual BTIC lines were plated at one million cells/ml, after 48 hrs media was collected and centrifuged to remove debris.

### CAD cell Media and EV collection

Media was collected 48 hrs after transfection unless otherwise indicated and spun at 1,500Xg for 10 min to remove cell debris. Media was concentrated 10Xs with YM 30,000, centricon filter (Millipore) at 5,000Xg for 30 min. Cells were lysed in: 40 mM Tris (pH7.4), 150 mM NaCl, 2 mM EDTA, 1 mM EGTA, 1% Triton X-100, 0.1% SDS with 1 mM Na_3_VO_4_, 0.5 mM PMSF and protease inhibitors (Sigma) at 4 °C for 1 hr. Lysates were centrifuged at 15000Xg for 5 min at 4° C and the supernatant (soluble fraction) was collected and stored at −70° C. Protein concentration of the soluble CAD cell lysate was determined using the Pierce BCA protein assay.

For isolation of EV’s, media was harvested 48 hrs after transfection, spun at 2000Xg for 10 min to remove cell debris and the supernatant was combined 5:1 with exoquick precipitation solution (SBI), incubated overnight at 4 °C, centrifuged at 7,000Xg for 30 min, washed and resuspended in PBS.

### Immunoblotting

Proteins were separated by SDS-PAGE and electrotransferred from polyacrylamide gels to nitrocellulose membrane (0.2 µm pore size). Membranes were blocked in tris-buffered saline (TBS) containing 0.1% Tween 20, 1% BSA and then incubated with primary antibody overnight at 4° C. The membranes were washed and incubated with horseradish peroxidase-coupled secondary antibody for ~2 hrs at room temperature. Bound antibodies (Supp Table 1) on the membranes were detected by incubation with Pierce chemiluminescent reagent and exposure to Cdigit, LiCor (Mandel). The chemiluminescent signals were quantified using image studio digits software (Mandel).

### EV size determination

Nanosight finite track length analysis was used to determine the EV size distribution (System Biosciences).

### Brain Slices

All mice were maintained in accordance with an animal protocol approved by the University of Calgary and the Guidelines for Lab Animal Safety. All methods were conducted in accordance with the relevant University of Calgary guidelines and regulations. We isolated EV’s from the brains of 23–25 day old WT and littermate CSPα knock out mice by a procedure modified from ref. 85. Mouse brains were removed, separated into hemi brains and the hemi brain sliced into four pieces and incubated for 2 hrs at 37 °C in 6 ml of hibernate A. Media was filtered through a 30 µm filter, centrifuged 2,000Xg for 10 min, the supernatant combined 5:1 with exoquick precipitation solution (SBI), incubated overnight at 4 °C, centrifuged at 7,000Xg for 30 min and washed and resuspended in PBS. For preparation of EV’s by differential ultracentrifugation, media was filtered through a 30 µm filter and sequentially at centrifuged 2,000Xg for 10 min, and at 10,000Xg for 30 min at 4 °C to discard membranes and debris. The supernatant was then centrifuged at 100,000Xg for 2 hrs to pellet EV’s enriched in exosomes.

### Statistical Analysis

All values are presented as the mean ± SEM and statistical significance was analyzed using a Student’s t-test. Calculations were performed using GraphPad Prism 6 software.

## Electronic supplementary material

Supplementary Table 1
