# Supplementary material for: Neurons Export Extracellular Vesicles Enriched in Cysteine String Protein and Misfolded Protein Cargo
Source: Sci Rep. 2017 Apr 19;7:956. doi: 10.1038/s41598-017-01115-6 (PMC5430488; doi:10.1038/s41598-017-01115-6)
Supplement: Supplementary file 1 — Supplementary Table 1 [file 41598_2017_1115_MOESM1_ESM.pdf]

Neurons Export Extracellular Vesicles Enriched in Cysteine String Protein and Misfolded Protein Cargo

Jingti Deng, Carolina Koutras, Julien Donnelier, Mana Alshehri, Maryam Fotouhi, Martine Girard, Steve Casha, Peter S. McPherson, Stephen M. Robbins, Janice E. A. Braun

Supplementary Table 1. Antibodies used in this Study

Anti CSP polyclonal	(Braun and Scheller, 1995)
Anti-myc monoclonal	Clonetech laboratories Inc.
Anti-FLAG monoclonal	Clonetech laboratories Inc.
Anti-actin monoclonal	Sigma-Aldrich
Anti-Hsp70/Hsc70 monoclonal	Sigma-Aldrich
Anti-clathrin monoclonal	BD Transduction Labs
Anti-flotillin-1 monoclonal	BD Transduction Labs
Anti-Hsp90 polyclonal	StressMarq Biosciences
Anti-DnaJB1 polyclonal	Thermo Fisher Scientific
Anti-DnaJB11 polyclonal	Cedarlane
Anti-DnaJA1 monoclonal	Cedarlane
Anti-$G_{\alpha s}$ polyclonal	Santa Cruz Biotechnology
Anti-SNAP25 monoclonal	Sternberger Monoclonals Inc.
Anti-BK monoclonal	BD Transduction Labs
Anti syntaxin monoclonal	Sigma-Aldrich
Anti-synaptotagmin polyclonal	Sigma-Aldrich
Anti-dynamin monoclonal	BD Transduction Labs
Anti-GFP polyclonal	Santa Cruz Biotechnology
Anti-ILK monoclonal	Ab Frontier
Anti-SOD1 polyclonal	Enzo
Anti-huntingtin monoclonal	Millipore
